# Behavioral Disruption in *Brachionus plicatilis* Exposed to Bisphenol A: A Locomotion-Based Assessment

**DOI:** 10.3390/toxics13090723

**Published:** 2025-08-28

**Authors:** Quang-Anh Tran, Nhat-Truong Phan, Quynh-Anh Tran-Nguyen, Hong Thi Mai, Thao Linh Thi Phan, Mau Trinh-Dang

**Affiliations:** 1 Environment & Biological Resource (DN-EBR), University of Science and Education, 459 Ton Duc Thang St., Danang 550000, Vietnammaithihongb9@gmail.com (H.T.M.);; 2 Faculty of Biology, Agriculture, and Environmental Science, The University of Da Nang—University of Science and Education, 459 Ton Duc Thang St., Danang 550000, Vietnam

**Keywords:** rotifer, bisphenol A, swimming behavior, concentration time response, *Brachionus plicatilis*

## Abstract

This study investigates the effects of Bisphenol A (BPA)—a ubiquitous endocrine disruptor—on the swimming behavior of the rotifer *Brachionus plicatilis*. Across a 0–40 ppm gradient, a biphasic response was observed, with swimming speed peaking at 20 ppm (100.42 ± 12.17 µm/s) and then significantly declining by 43% to 57.58 ± 30.59 µm/s at 40 ppm (Tukey, *p* < 0.05). Speed–frequency plots revealed co-existing hyper- and hypoactive sub-populations at 10–30 ppm, whereas severe inhibition dominated at 40 ppm. Additionally, temporal analysis confirmed that BPA effects were both concentration- and time-dependent, with the mean speed at 10 ppm declining only slightly over time (slope ≈ −0.8), whereas at 40 ppm, the decrease was an order of magnitude steeper (slope ≈ −16.9). Additionally, BPA exposure also triggered a sharp rise in abrupt turns (582.53 ± 477.55 events) and greater path sinuosity, consistent with neuromuscular disturbance. These findings demonstrate that rotifer locomotion provides an early and sensitive indicator of environmental BPA exposure.

## 1. Introduction

Behavior is a fundamental biological response that integrates physiological and environmental cues to regulate an organism’s interaction with its surroundings. As such, behavioral alterations often serve as early indicators of environmental stress, preceding detectable physiological or biochemical disruptions [1]. Among behavioral endpoints, locomotion is particularly relevant in aquatic ecosystems, as it underpins critical ecological functions such as foraging, predator avoidance, and reproductive success [2]. Recent advancements in ecotoxicology have highlighted swimming behavior as a highly sensitive metric for assessing pollutant-induced neurotoxicity, offering rapid and quantifiable insights into sublethal stress responses [3,4].

Rotifers (*Brachionus* spp.) are key components of aquatic food webs, transferring energy from microbial producers to higher trophic levels [5]. Their short generation time, high fecundity, and ease of laboratory cultivation have made them valuable model organisms in ecotoxicology [4,6]. Specifically, *Brachionus plicatilis* has emerged as a suitable bioindicator for aquatic pollution, exhibiting quantifiable behavioral and physiological responses to various environmental contaminants, including heavy metals, endocrine-disrupting chemicals, and nanoplastics [7,8]. Given that rotifer locomotion is closely linked to essential ecological functions such as conspecific recognition, mating, and feeding, any impairment in swimming ability may disrupt trophic interactions, resource acquisition, and ultimately population stability [9]. Therefore, behavioral analysis of rotifer swimming patterns provides a powerful tool for detecting sublethal toxic effects in aquatic ecosystems.

Among the numerous pollutants threatening aquatic environments, bisphenol A (BPA) is of particular concern due to its widespread use in polycarbonate plastics and epoxy resins. BPA enters aquatic systems primarily through industrial discharge and plastic waste degradation, with environmental concentrations ranging from nanograms to micrograms per liter [10,11]. As an endocrine-disrupting chemical (EDC), BPA mimics endogenous hormones, interfering with estrogenic pathways and causing developmental, metabolic, and reproductive disruptions across a wide range of taxa [12]. Its toxicological effects on aquatic organisms have been well documented, with studies demonstrating BPA-induced oxidative stress, reproductive impairment, and altered metabolic activity in diverse species, including fish, crustaceans, and rotifers [13,14,15].

Although BPA has been implicated in neurotoxicity, its specific effects on rotifer locomotion remain poorly understood. Existing studies have primarily focused on reproductive and metabolic endpoints [14,15], while behavioral disruptions, which may serve as an early and functionally significant indicator of toxicity, have received less attention. Rotifers possess a simple yet functionally sophisticated nervous system capable of mediating complex motor responses [5] and provide a suitable model for evaluating BPA-induced neuromuscular impairments. Furthermore, BPA exposure has been linked to oxidative stress and disruptions in neural signaling pathways, which could plausibly contribute to altered locomotor dynamics [15].

This study investigates the impact of BPA on rotifer swimming behavior by quantifying alterations in key locomotor parameters, including swimming speed, trajectory complexity, and directional stability. By integrating behavioral assessments with toxicological analysis, we aim to advance the understanding of BPA-induced neurophysiological effects in planktonic rotifers. Our findings will contribute to a more comprehensive ecological risk assessment of BPA and underscore the value of behavioral endpoints in environmental monitoring.

## 2. Materials and Methods

### 2.1. Materials

**Figure 1:**
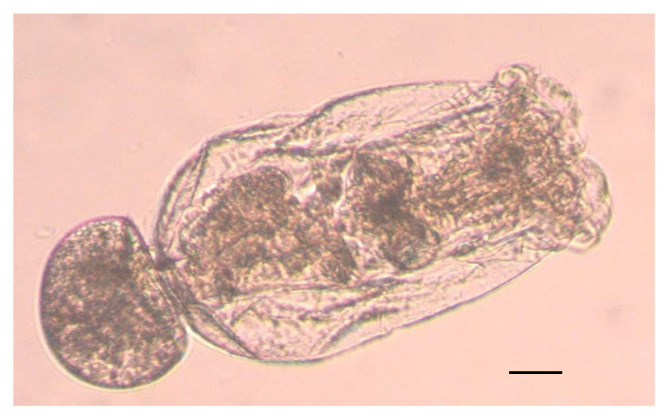
*Brachionus plicatilis* Koste, 1978 (photographs by the authors).

The monogonont rotifer *Brachionus plicatilis* was isolated from water samples collected at the Han River, Da Nang City, Vietnam. Species identification was confirmed using the taxonomic key by Koste (1978) [16] (Figure 1). For laboratory culture, rotifers were maintained in filtered artificial seawater. The strain was preserved by serial transfer of asexual populations at 25 °C under a 12:12 h light–dark photoperiod with 10 practical salinity units (psu) of salinity. The green algae *Chlorella vulgaris* were used as a live diet (approximately 6 × 10^5^ cells/mL), was provided by the Algal Biotechnology Laboratory, The University of Danang, Danang, Vietnam.

Bisphenol A (BPA) has the chemical formula (CH_3_)_2_C(C_6_H_4_OH)_2_, a molecular weight of 228.29 g/mol, and a purity of ≥ 99% (Sigma-Aldrich, St. Louis, MO, USA). This compound was dissolved in dimethyl sulfoxide (DMSO), ensuring that the solvent concentration in the exposure groups did not exceed 0.1% DMSO (Scientific and Technical Laboratory Co., Ltd., Ho Chi Minh City, Vietnam). The dilution process was conducted following standard methodologies to ensure precision and reproducibility.

### 2.2. Experimental Design

A controlled experimental setup was used to evaluate the impact of Bisphenol A (BPA) on the movement behavior of rotifers. BPA was tested at concentrations of 0, 10, 20, 30, and 40 ppm. These concentrations were chosen based on preliminary acute LC_50_ values (e.g., 12 h LC_50_ = 18.99 mg L^−1^), ensuring a gradient from sublethal to near-lethal stress for observing behavioral effects without confounding mortality, as selecting sub- to near-threshold levels is standard practice when the endpoint is behavior rather than mortality [17]. All treatments, including the 0 ppm control, contained 0.1% DMSO to maintain consistency in solvent exposure, with three replicates for each treatment. In total, 15 rotifers per treatment were tracked (5 individuals per replicate × 3 replicates). A 20 µL test solution containing BPA diluted in culture medium was gently introduced into each microwell of the experimental chamber. Rotifers were simultaneously exposed under identical environmental conditions but were individually confined within separate wells to eliminate physical interaction and ensure uniform exposure across individuals. The environmental conditions were maintained at a temperature of 25 °C, salinity of 10 ppt, and illumination intensity of 700 lux to ensure uniform testing conditions and minimize confounding factors. Exposure lasted exactly 5 min, a duration chosen to capture acute behavioral responses while minimizing secondary effects. Behavioral tracking was conducted in real time during exposure, with video recording initiated immediately upon BPA addition.

### 2.3. Rotifer Locomotion Behavior Tracking and Analysis

**Figure 2:**
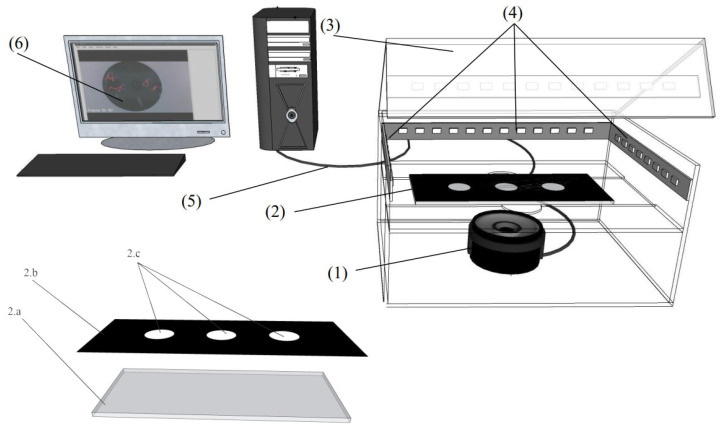
Schematic diagram of the rotifer behavior tracking system (top, 1–6) and the structure of the chamber (bottom, 2a, 2b, 2c).

The experimental setup was constructed as a closed observation system, offering a controlled environment for monitoring rotifer behavior under optimal conditions (Figure 2). A centrally positioned camera (1) beneath the observation chamber (2) within a sealed enclosure (3) minimizes external light interference. The chamber used for rotifer observation (2) consists of three main components: a transparent glass plate (2a), a black waterproof decal sheet (2b), and microwells (2c). The glass plate (2a) serves as the base of the chamber, providing an optically clear surface that facilitates high-resolution imaging from below. Mounted on top is the black decal sheet (2b), which reduces ambient light reflection and enhances contrast between the rotifer and the background, thereby improving image quality during tracking. Wells (2c) are precisely cut into this decal sheet, each measuring 3.5 mm in diameter and 0.076 mm in depth, forming shallow individual arenas. Each well houses five adult rotifers in a contained microenvironment. The modular design allows flexible adjustments to well number and size while reducing ambient light exposure. An active lighting system (4), integrated with the sealed cover, stabilizes illumination for precise tracking. The camera connects via cable (5) to a computer, where the video feed is displayed on a monitor (6) for real-time observation.

Rotifer movement in each triplicate sample was recorded at 20 frames per second using a high-speed digital camera (OV3660 Camera Module, 3MP, USB, 110° wide-angle, 2.1 mm lens). Twenty XY coordinates of the animal’s barycenter were extracted per second and stored for subsequent analysis. Swimming speed (µm/s) was calculated as the displacement between two consecutive frames. To assess locomotor patterns, both speed distribution histograms and temporal variations in speed were examined. Movement angles (°) were derived from three successive positions, and abrupt directional changes were quantified by counting instances where turning angles exceeded 120°. Additionally, the sinuosity index, reflecting path curvature, was calculated based on angular deviations (in radians) between angles β and α, following the method of Kagali et al. (2023) [18].

All tracking data were compiled into a single dataset and analyzed using Google Colab. For all inferential analyses, individual trajectories within a replicate were averaged to yield a single replicate-level value, and statistical tests were conducted on the three replicate means (*n* = 3 per treatment). The distribution plot of instantaneous speeds is shown for visualization only and is constructed by pooling all timepoints and individuals within each treatment; no statistical inference is drawn from this plot. A one-way ANOVA was conducted to assess significant differences in mean swimming speed and the frequency of abrupt directional changes between the control and treatment groups. Where statistical significance was observed, a Tukey’s post hoc test was applied to correct for multiple comparisons. All statistical analyses were performed at a significance level of *p* < 0.05.

## 3. Results

### 3.1. Average Swimming Speed

**Figure 3:**
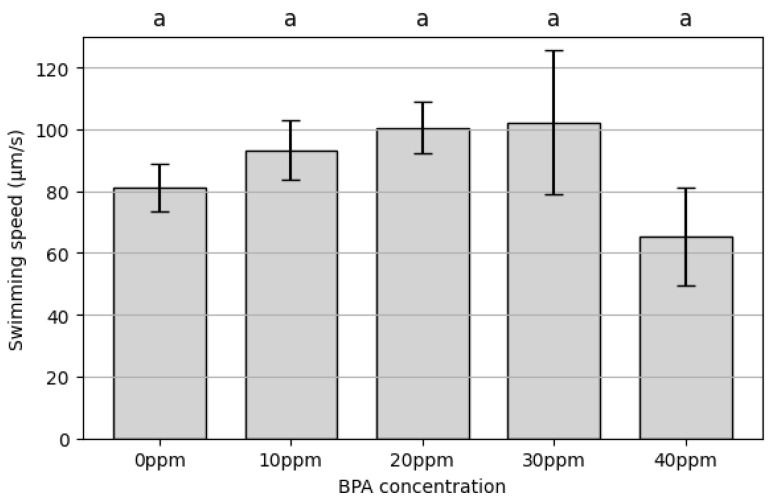
The average swimming speed of rotifers in different BPA concentrations. Data are presented as mean ± SD (*n* = 3 replicates). Columns sharing the same superscript letter are not significantly different (Tukey HSD, α = 0.05).

Figure 3 illustrates the variation in average swimming speed of rotifers across different BPA concentrations. Swimming speed showed a slight increase from 10 ppm to 30 ppm, peaking at 102.14 ± 23.26 µm/s at 30 ppm. Notably, standard deviations were larger at higher BPA concentrations, indicating greater variability in inter-individual responses to toxicity. At 40 ppm, swimming speed sharply declined to 65.29 ± 15.69 µm/s. A one-way ANOVA detected a significant overall treatment effect (F(4,10) = 3.51, *p* = 0.04877). However, Tukey’s HSD controlling the familywise error at 0.05 did not identify any pairwise differences; the 30 vs. 40 ppm contrast showed a near-significant trend (*p* = 0.0595) (Table S1). These results suggest a biphasic response to BPA, with a slight, non-significant elevation in motility at lower BPA concentrations followed by a decline at the highest concentration.

### 3.2. Speed Distribution

**Figure 4:**
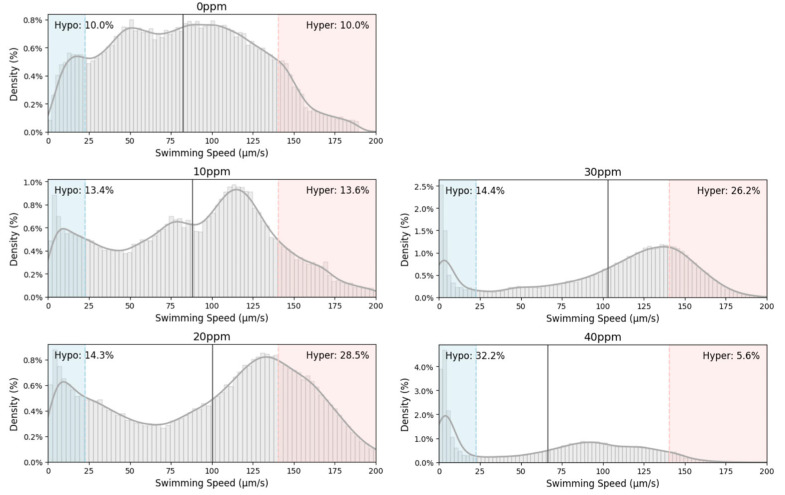
Speed distribution of rotifers in different BPA concentrations. (Light gray bars show density (%); the dark gray curve is the kernel density estimation; light blue and light red shading mark speeds below the 10th and above the 90th percentile of the 0 ppm control, respectively; inset percentages report the proportion of hypo- and hyperactive observations; the solid black line indicates the mean speed).

The speed distribution analysis provides deeper insight into the behavioral response of rotifers to BPA exposure (Figure 4).

In the control group (0 ppm), swimming speeds were normally distributed around 77 µm/s with low variation, indicating consistent swimming behavior. At 10 ppm, the highest density shifted to ~120 µm/s, suggesting increased activity in a subset of rotifers. Notably, a bimodal response emerged: 13.4% of observations were hypoactive (<20 µm/s) and 13.6% hyperactive (>150 µm/s), both higher than in controls, indicating divergent individual responses. This divergence widened at 20 ppm, where the low-speed peak narrowed to 5–10 µm/s and the high-speed peak moved to 130–140 µm/s; the hyperactive fraction rose to 28.5% while hypoactivity remained at 14.4%. In the 30 ppm treatment, variability persisted: although hypo- and hyperactivity percentages were similar to that of the 20 ppm treatment, the proportion of speeds < 10 µm/s markedly increased, suggesting more severe locomotor impairment. At 40 ppm, the distribution collapsed toward minimal movement: 32.2% of data points lay below 20 µm/s, and only 5.6% exceeded 150 µm/s, reflecting near-complete locomotor suppression. Collectively, these profiles demonstrate that sublethal BPA exposures (10–30 ppm) provoke both overstimulation and inhibition of rotifer swimming, whereas higher concentrations overwhelmingly drive the population into a hypoactive state.

### 3.3. Temporal Change in Speed

**Figure 5:**
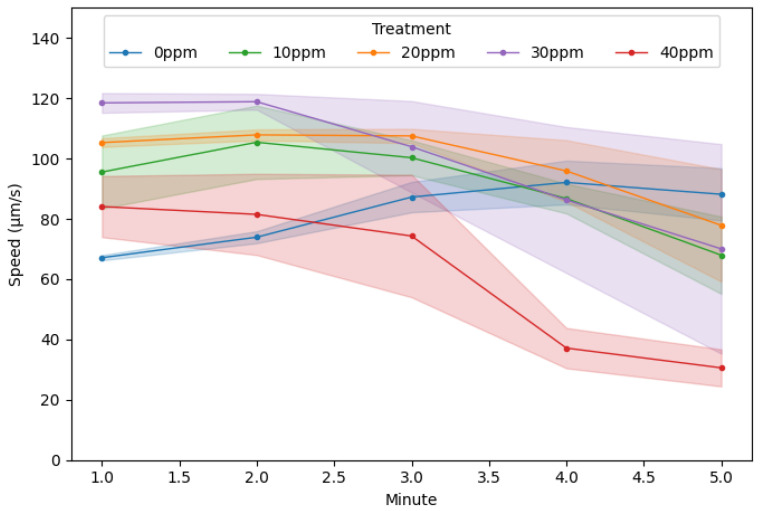
Temporal dynamics of swimming speed of rotifers in different BPA treatments. (Solid circles (●): per-minute mean speed; shaded bands: standard error of the mean).

In the control group (0 ppm), mean swimming speed exhibited a slight upward trend over the five-minute observation period, whereas all BPA-exposed treatments showed net declines (Figure 5). Remarkably, in the first minute, every BPA concentration exceeded the control, with the 20 ppm (105.31 ± 1.45 µm/s) and 30 ppm (118.48 ± 3.27 µm/s) groups swimming significantly faster than all others (*p* < 0.05), indicating an acute hyperactive response upon initial toxin exposure. This elevated locomotion generally persisted through the second and third minutes; thereafter, all BPA treatments—especially 40 ppm—underwent steep speed reductions during the fourth and fifth minutes. In particular, mean speed in the 40 ppm group fell from 84.09 ± 10.13 µm/s in the first minute to 30.59 ± 6.14 µm/s in the fifth minute, revealing a rapid onset of locomotor impairment at high concentration (Table 1 and Table S2).

Acceleration data mirrored these trends: control rotifers accelerated modestly (5.26 ± 1.99 µm/s per min), whereas BPA-exposed groups decelerated in a concentration-dependent manner (10 ppm: −6.90 ± 6.07; 20 ppm: −6.86 ± 4.30; 30 ppm: −12.11 ± 8.21; 40 ppm: −14.25 ± 4.62 µm/s per min). Altogether, these results reveal a biphasic response—initial stimulation followed by progressive locomotor decline—with high BPA concentrations inducing rapid and severe movement impairment.

### 3.4. Moving Behavior Through Abrupt Directional Change and Sinuosity

**Figure 6:**
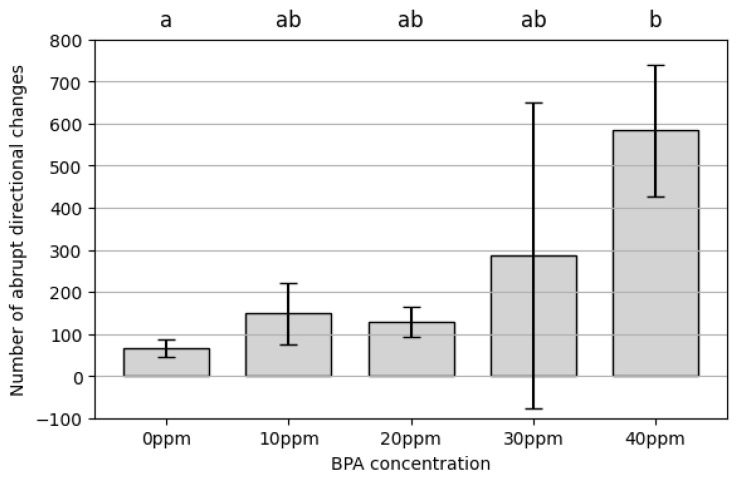
Number of abrupt directional changes of rotifers in different treatments. Data are presented as mean ± SD (*n* = 3 replicates). Columns sharing the same superscript letter are not significantly different (Tukey HSD, α = 0.05). The large SD at 30 ppm is a result of high variability between replicates, with individual values of 145.3, 15.6, and 700.8 turns.

Quantification of abrupt directional changes further reinforces the observed locomotor instability. The mean number of turns exceeding 120° moderately increased at 10 ppm (148.27 ± 72.58 times) and 20 ppm (128.60 ± 35.92 times) but sharply rose at 30 ppm (287.20 ± 363.96 times) and 40 ppm (583.44 ± 157.58 times), indicating severe disruptions in movement control (Figure 6). The large standard deviations at 30 ppm and 40 ppm suggest highly variable individual responses, with some rotifers displaying extreme movement instability. ANOVA results indicated significant differences among treatments (F(4, 10) = 3.91, *p* = 0.036), and Tukey’s HSD identified a significant difference only between 40 ppm and the control (0 ppm) (*p* = 0.0362); all other pairwise comparisons were not significant (Table S3).

**Figure 7:**
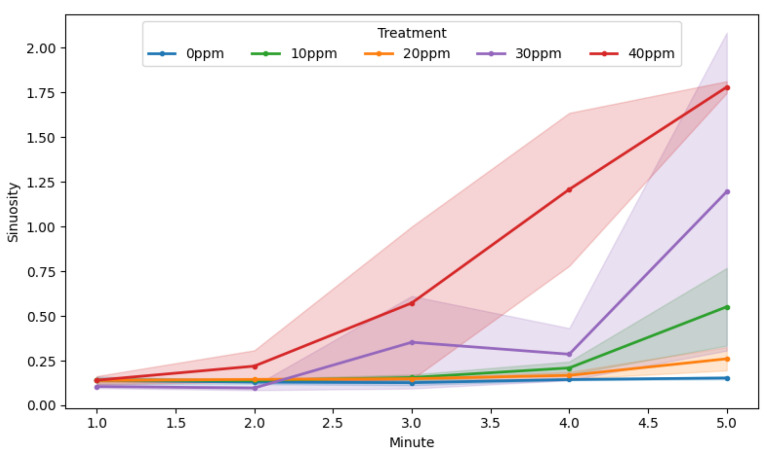
Sinuosity of rotifers in different treatments during the observation. (Solid circles (●): per-minute mean sinuosity; shaded bands: standard error of the mean).

BPA exposure altered rotifer movement trajectories in a clear, concentration- and time-dependent manner (Figure 7, Table 2). Under control conditions, sinuosity remained low and stable (~0.14 ± 0.021) throughout the five-minute observation, reflecting smooth, directed swimming. At 10 ppm and 20 ppm, mean sinuosity rose modestly—reaching 0.551 ± 0.217 and 0.26 ± 0.065 by Minute 5—but did not significantly differ from the control. In the 30 ppm group, rotifers displayed more erratic paths (0.353 ± 0.259 at Minute 3; 1.195 ± 0.889 at Minute 5), though high inter-individual variability precluded statistical separation from lower concentrations (Figure S1). By contrast, 40 ppm elicited a pronounced and significant surge in sinuosity beginning at Minute 4 (1.208 ± 0.428 vs. 0.144 ± 0.005 in control; Tukey HSD, *p* < 0.05) and persisting at Minute 5 (1.780 ± 0.019) (Table S4). Together with the increase in abrupt directional changes, these results demonstrate that high BPA levels progressively disrupt locomotor coordination, producing highly erratic swimming paths.

## 4. Discussion

### 4.1. Concentration-Dependent Response in Swimming Behavior

Our study demonstrates that BPA exposure induces a non-monotonic, concentration-dependent alteration in the swimming behavior of the rotifer *B. plicatilis*. Moderate exposure (~30 ppm) elicited a non-significant upward trend in swimming speed, increasing by approximately 35%, *p* > 0.05 (Figure 3). This transient stimulation likely reflects a compensatory physiological response to mild toxic stress, consistent with prior research suggesting that aquatic invertebrates can upregulate metabolic activity as an adaptive strategy to cope with predation threats and environmental contaminants [19,20]. In contrast, a higher concentration (40 ppm) depressed locomotor performance below baseline (Figure 3). This biphasic response, also known as hormesis, has been observed in other organisms exposed to BPA and other endocrine-disrupting chemicals (EDCs) [21,22].

Drawing on extensive mechanistic studies, locomotor impairment at high BPA concentrations arises from the convergence of multiple physiological and neurotoxic pathways that disrupt the neurotransmitter systems and ion channels governing movement, as presented in Table 3. At the synaptic level, BPA targets the cholinergic axis: in a variety of aquatic organisms, it inhibits acetylcholinesterase (AChE) activity, which allows acetylcholine to accumulate at neuromuscular junctions, producing an initial burst of excitation that soon devolves into fatigue and, at sufficiently high exposures, paralysis [23,24,25]. Furthermore, while direct evidence from small crustaceans remains scarce, electrophysiological studies in larger organisms show that BPA can also directly influence the function of ion channels by blocking voltage-activated Ca^2+^ channels (L, N, P/Q, T, R types) with comparable potency, exhibiting EC50 values ranging from 26 to 35 μM, and modulating K^+^ channels, causing altered neuronal excitability and impaired muscle contraction [26,27].

### 4.2. Time-Dependent Changes in Swimming Behavior

Beyond concentration effects, our results indicate that exposure duration significantly shapes motility responses. Temporal analysis revealed an initial increase in speed followed by a subsequent decline, suggesting energy depletion and progressive impairment within a short timeframe (≤5 min). Simultaneously, sinuosity increased over time, particularly at higher concentrations, indicating a shift toward more erratic and uncoordinated movement patterns. This behavioral sequence aligns with Gerhardt’s Stepwise Stress Model (SSM), which describes a phased response to toxicants—organisms initially exhibit hyperactivity (resistance phase) before transitioning to hypoactivity (regulatory phase) as physiological stress accumulates [30]. Similar patterns were reported in *Gammarus pulex*, where initial hyperactivity was followed by reduced movement at higher toxicant levels [31], reinforcing the role of exposure duration as a critical factor in shaping behavioral outcomes.

Evidence from earlier studies leads us to hypothesize that the progressive loss of mobility and orientation observed may stem from BPA-induced neuromuscular disruption and accompanying oxidative stress [32,33]. BPA can increase the production of reactive oxygen species (ROS), leading to redox imbalance and cellular damage [15]. In the crayfish *Astacus leptodactylus*, BPA exposure increased the activity of antioxidant enzymes like SOD and GPx but decreased GR activity while increasing MDA levels (a marker of lipid peroxidation) [25]. The resulting ROS-mediated oxidative stress in high-metabolic-rate neuronal and muscular tissues is therefore expected to compromise neuromuscular performance and mechanistically underpins the locomotor deficits observed after BPA exposure [34]. Furthermore, BPA can interfere with cellular energy metabolism by affecting the activity of crucial ATPases [25] and the expression of genes involved in lipid metabolism [15], potentially leading to locomotor fatigue and reduced ability to sustain swimming activity over time. Further supporting this, exposure to bromate has been linked to oxidative stress and motility impairment, with reductions in antioxidant activity (e.g., glutathione), increasing susceptibility to cellular damage [35]. These findings suggest that oxidative stress and neuromuscular disruption may be key mechanisms underlying BPA-induced locomotor impairments, consistent with observations of erratic movement and hypoactivity at higher concentrations.

The potential for recovery of locomotor function after the cessation of BPA exposure is a critical aspect not addressed in this study, but it has significant ecological implications. If the effects are irreversible or the recovery is slow, even a temporary exposure to BPA could result in long-term negative effects on rotifer populations.

### 4.3. Erratic Movement as a Marker of Toxicant Exposure

A striking behavioral alteration observed in our study was a sharp increase in abrupt directional changes (>120°) at higher BPA concentrations, with frequencies rising by less than twofold at 10–20 ppm but escalating to roughly four- to sixfold above control levels at 30–40 ppm. This pattern of erratic movement is consistent with prior findings on toxicant-induced swimming irregularities, where pollutants such as copper, pentachlorophenol, and bromate caused increased movement instability, impaired directional control, and reduced overall swimming capacity of *B. calyciflorus* and *B. plicatilis* [36].

Erratic movements and the tendency to swim in small, repetitive circular patterns were particularly evident at high BPA concentrations. This behavior has been described in previous studies, where rotifers exhibited confined, disoriented movement before eventually losing motility [37]. These movement patterns are often associated with neuromuscular dysfunction or energy depletion, marking the transition from hyperactivity to hypoactivity and impaired physiological function [17,38]. Consistent with this transition, studies in other aquatic vertebrate models—particularly zebrafish and goldfish—have documented that BPA elicits a broader neurotransmitter disequilibrium beyond cholinergic perturbation, notably involving GABAergic imbalance (decreased GABA with concomitant increases in glutamate/glutamine) and multiple dopaminergic alterations, together with pronounced oxidative stress responses [39]. In another study on *Caenorhabditis elegans*, BPA has been shown to damage serotonergic, dopaminergic, and GABAergic neurons and alters related mRNA expression [26]. Given the phylogenetically conserved roles of these neurotransmitter systems in locomotor regulation, it is plausible that similar multi-system disruptions, along with oxidative stress, contributed to the complex and erratic movement patterns we observed in rotifers.

The ecological implications of these findings are significant, as swimming behavior is essential for predator avoidance, resource acquisition, and reproductive success in zooplankton. Specifically, changes in swimming behavior, such as speed, path complexity, or reaction time, may make *B. plicatilis* more vulnerable to predation. For instance, pesticides have been shown to induce maladaptive responses to predator chemical cues in zooplankton [40]. Moreover, impaired locomotion can reduce rotifers’ ability to encounter and ingest food particles, adversely affecting their growth and reproduction. Additionally, the mating success of zooplankton or sexually reproducing species may also be significantly impacted, as changes in locomotion may reduce mate encounter rates, affecting fertilization and resting egg production, which are important for population survival.

Our trajectory analysis provides high-resolution insights into BPA-induced behavioral modifications, capturing subtle, dynamic changes that conventional toxicity endpoints (e.g., mortality, reproductive success) might overlook. The near-complete movement restriction observed at 40 ppm likely represents a critical neurotoxic threshold with potential ecological consequences. These detailed behavioral assessments extend the findings of Fabrello et al. (2022) [41], demonstrating the importance of tracking locomotor responses over time to fully characterize toxicant impacts. Additionally, our findings challenge the traditional assumption of a linear exposure–response model, as BPA induced both stimulatory and inhibitory effects depending on concentration and duration of exposure. This highlights the need for more sophisticated toxicological assessment frameworks that account for non-monotonic concentration–response relationships, hormetic effects, and inter-individual variability. Importantly, the occurrence of erratic movement, often preceding more severe outcomes, may offer a sensitive and quantifiable behavioral biomarker for environmental monitoring. By incorporating such early-warning behavioral indicators, monitoring programs can detect sublethal toxicant effects more effectively and respond before ecological damage becomes irreversible. Future studies employing transcriptomic and metabolomic approaches will be crucial in elucidating the molecular mechanisms underlying these behavioral disruptions.

## 5. Conclusions

This study demonstrates that BPA exposure induces concentration- and time-dependent disruptions in rotifer locomotion. At concentrations of 10 to 30 ppm, both hyperactivity and reduced motility were observed, resulting in heterogeneous behavioral responses despite no significant change in average speed. At 40 ppm, movement was severely impaired, with many individuals exhibiting near immobility, indicating progressive neuromotor dysfunction. Speed distribution and temporal analysis further revealed that BPA exposure led to increasingly erratic movement, loss of directional control, and widespread inhibition at higher concentrations. These findings suggest that BPA initially triggers variable locomotor responses at lower concentrations before causing severe motor suppression at higher concentrations. The observed behavioral disruptions, particularly at 40 ppm, indicate potential neurotoxic effects. Further research is needed to elucidate the mechanisms underlying these behavioral alterations. Importantly, these behavioral endpoints hold promise as sensitive biomarkers for detecting sublethal toxicant effects in environmental monitoring.

## Figures and Tables

**Table 1:** Summary of mean ± SD swimming speed by minute (µm/s) and acceleration (µm/s per min) (superscript letters denote groups not significantly different at each minute (Tukey HSD, α = 0.05)).

Treatment	Min 1	Min 2	Min 3	Min 4	Min 5	Acceleration
0 ppm	67.12 ± 1.52 ^a^	73.93 ± 3.44 ^a^	87.24 ± 8.73 ^a^	92.10 ± 12.56 ^a^	88.16 ± 15.02 ^a^	5.26 ± 1.99
10 ppm	95.54 ± 21.01 ^abc^	105.42 ± 21.07 ^ab^	100.28 ± 10.02 ^a^	86.74 ± 8.53 ^a^	67.96 ± 22.18 ^a^	−6.90 ± 6.07
20 ppm	105.31 ± 2.50 ^bc^	107.87 ± 3.14 ^ab^	107.52 ± 4.10 ^a^	95.90 ± 17.70 ^a^	77.86 ± 32.26 ^a^	−6.86± 4.30
30 ppm	118.48 ± 5.66 ^b^	118.87 ± 4.53 ^b^	103.94 ± 26.24 ^a^	86.28 ± 41.94 ^a^	70.06 ± 60.21 ^a^	−12.11 ± 8.21
40 ppm	84.09 ± 17.55 ^ac^	81.49 ± 23.33 ^ab^	74.32 ± 35.18 ^a^	37.15 ± 11.58 ^a^	30.59 ± 6.14 ^a^	−14.25± 4.62

**Table 2:** Summary of mean ± SD sinuosity by minute (superscript letters denote groups not significantly different at each minute (Tukey HSD, α = 0.05)).

Treatment	Min 1	Min 2	Min 3	Min 4	Min 5
0 ppm	0.140 ± 0.037 ^a^	0.130 ± 0.019 ^a^	0.127 ± 0.025 ^a^	0.144 ± 0.008 ^a^	0.153 ± 0.012 ^a^
10 ppm	0.142 ± 0.010 ^a^	0.139 ± 0.015 ^a^	0.156 ± 0.029 ^a^	0.209 ± 0.062 ^a^	0.551 ± 0.376 ^a^
20 ppm	0.139 ± 0.020 ^a^	0.145 ± 0.011 ^a^	0.149 ± 0.008 ^a^	0.168 ± 0.034 ^a^	0.260 ± 0.113 ^a^
30 ppm	0.105 ± 0.016 ^a^	0.097 ± 0.021 ^a^	0.353 ± 0.448 ^a^	0.286 ± 0.253 ^ab^	1.195 ± 1.540 ^a^
40 ppm	0.141 ± 0.040 ^a^	0.219 ± 0.153 ^a^	0.572 ± 0.741 ^a^	1.208 ± 0.741 ^b^	1.780 ± 0.033 ^a^

**Table 3:** Summary of known effects of BPA on key neurotransmitter systems and ion channels involved in the locomotion of certain species.

Target System/Molecule	BPA’s Effect	Species/Model System	Potential Consequences for Locomotion	References
Cholinergic system (AChE)	Inhibition of AChE activity	Ascidian *(Ciona robusta)*, date mussels (*Lithophaga lithophaga*), crayfish (*Astacus leptodactylus)*	Uncoordinated movement, hyperexcitability, muscle spasms, paralysis, altered activity levels	Melki et al., 2024 [23]; Abd Elkader & Al-Shami, 2023 [24]; Uçkun M., 2022 [25];
GABAergic system (GABA-A receptors)	Modulation of GABA-A receptors, neuronal damage, altered mRNA expression	Nematode *(Caenorhabditis elegans)*	Hyperexcitability or suppression of activity, impaired coordination, altered response to stimuli	Wang et al., 2023 [26]
Serotonergic system	Neuronal damage, Altered mRNA expression, altered neurotransmitter levels	Nematode (*C. elegans*), date mussels (*Lithophaga lithophaga*)	Changes in activity levels, altered arousal/responsiveness, altered path complexity (sinuosity)	Abd Elkader & Al-Shami, 2023 [24]; Wang et al., 2023 [26];
Glutamatergic system	Glutamatergic pathway upregulation, disrupted gene expression, excitation/inhibition (E/I) imbalance	Zebrafish (*Danio rerio*)	Reduced total distance and swimming speed, elevated anxiety-like behavior	Naderi et al., 2021 [28]
Voltage-gated Ca^2+^ channels (L, N, P/Q, T, R)	Blockade (EC50: 26–35 µM)	Rat—rat endocrine cells, mouse DRG neurons, cardiac myocytes, HEK293 cells	Reduced muscle contractility, impaired neurotransmitter release, and general locomotor impairment	Deutschmann et al., 2012 [29]
K^+^ channels (e.g., BK_Ca_)	Modulation (activation or inhibition, depending on channel and context)	Wistar rats—rat coronary smooth muscle cells	Altered neuronal excitability, altered muscle cell responsiveness, changes in rhythmic activity patterns	Costa et al., 2025 [27]

## Data Availability

The raw data supporting the conclusions of this article will be made available by the authors on request.

## Supplementary Materials

The following supporting information can be downloaded at: https://www.mdpi.com/article/10.3390/toxics13090723/s1, Table S1. Replicate-level mean (±SD) swimming speed by BPA treatment (0–40 ppm), with one-way ANOVA and Tukey HSD pairwise comparisons. Table S2. Pairwise Tukey HSD *p*-values for mean swimming speed comparisons between BPA treatments at each minute; Table S3. Replicate-level mean (±SD) number of abrupt directional changes by BPA treatment (0–40 ppm), with one-way ANOVA and Tukey HSD pairwise comparisons; Figure S1. Some trajectories of rotifer individuals under varying BPA exposure conditions; Table S4. Pairwise Tukey HSD *p*-values for sinuosity comparisons between BPA treatments at each minute.
