# Supplementary material for: Behavioral Disruption in Brachionus plicatilis Exposed to Bisphenol A: A Locomotion-Based Assessment
Source: Toxics. 2025 Aug 28;13(9):723. doi: 10.3390/toxics13090723 (PMC12473341; doi:10.3390/toxics13090723)

Supplementary

Table S1. Replicate-level mean (±SD) swimming speed by BPA treatment (0–40 ppm), with one-way ANOVA and Tukey HSD pairwise comparisons.

```
◆ ANOVA Results:
                  sum_sq    df         F    PR(>F)
C(Treatment)  2829.875223   4.0  3.511484  0.04877
Residual      2014.728897  10.0       NaN      NaN

◆ Tukey HSD Results:
 Multiple Comparison of Means - Tukey HSD, FWER=0.05
=====================================================
group1 group2 meandiff p-adj   lower    upper  reject
-----------------------------------------------------
  0ppm  10ppm  12.1478 0.8278  -25.994 50.2896  False
  0ppm  20ppm    19.24 0.4962 -18.9019 57.3818  False
  0ppm  30ppm   20.974  0.419 -17.1678 59.1158  False
  0ppm  40ppm -15.8724 0.6583 -54.0142 22.2694  False
 10ppm  20ppm   7.0922 0.9699 -31.0497  45.234  False
 10ppm  30ppm   8.8262  0.936 -29.3156  46.968  False
 10ppm  40ppm -28.0202 0.1875  -66.162 10.1216  False
 20ppm  30ppm    1.734 0.9999 -36.4078 39.8758  False
 20ppm  40ppm -35.1124 0.0749 -73.2542  3.0295  False
 30ppm  40ppm -36.8464 0.0595 -74.9882  1.2954  False
-----------------------------------------------------

◆ Mean ± SD for each Treatment group:
  Treatment  Mean Speed   SD Speed
0      0ppm   81.167988   7.712420
1     10ppm   93.315778   9.544230
2     20ppm  100.407943   8.325118
3     30ppm  102.141962  23.261412
4     40ppm   65.295577  15.696809
```

Table S2. Pairwise Tukey HSD p-values for mean swimming speed comparisons between BPA treatments at each minute

**Treatment1**	**Treatment2**	**p_value. Min 1**	**p_value. Min 2**	**p_value. Min 3**	**p_value. Min 4**	**p_value. Min 5**
**0ppm**	**10ppm**	0.112	0.126	0.932	0.998	0.939
**0ppm**	**20ppm**	***0.026***	0.092	0.748	1	0.995
**0ppm**	**30ppm**	0.004	***0.022***	0.852	0.997	0.958
**0ppm**	**40ppm**	0.5	0.964	0.934	0.073	0.276
**10ppm**	**20ppm**	0.87	1	0.992	0.985	0.995
**10ppm**	**30ppm**	0.243	0.779	0.999	1	1
**10ppm**	**40ppm**	0.795	0.314	0.56	0.114	0.647
**20ppm**	**30ppm**	0.707	0.876	1	0.982	0.998
**20ppm**	**40ppm**	0.304	0.238	0.342	***0.052***	0.445
**30ppm**	**40ppm**	***0.046***	0.059	0.443	0.119	0.603

Table S3. Replicate-level mean (±SD) number of abrupt directional changes by BPA treatment (0–40 ppm), with one-way ANOVA and Tukey HSD pairwise comparisons.

```
 • ANOVA Results:
                   sum_sq    df         F    PR(>F)
C(Group)  514234.431407   4.0  3.912082  0.036494
Residual  328619.416852  10.0       NaN       NaN

 • Tukey HSD Results:
  Multiple Comparison of Means - Tukey HSD, FWER=0.05
========================================================
group1 group2 meandiff p-adj    lower     upper   reject
--------------------------------------------------------
  0ppm  10ppm  82.7333 0.9782 -404.3909  569.8575  False
  0ppm  20ppm  63.0667  0.992 -424.0575  550.1909  False
  0ppm  30ppm 221.6667 0.5859 -265.4575  708.7909  False
  0ppm  40ppm 517.9111 0.0362   30.7869 1005.0353   True
 10ppm  20ppm -19.6667 0.9999 -506.7909  467.4575  False
 10ppm  30ppm 138.9333 0.8755 -348.1909  626.0575  False
 10ppm  40ppm 435.1778  0.086  -51.9464   922.302  False
 20ppm  30ppm    158.6 0.8168 -328.5242  645.7242  False
 20ppm  40ppm 454.8444 0.0701  -32.2798  941.9686  False
 30ppm  40ppm 296.2444  0.331 -190.8798  783.3686  False
--------------------------------------------------------

 • Mean ± SD for each group:
   Group  Mean Count    SD Count
0   0ppm   65.533333   21.205031
1  10ppm  148.266667   72.579428
2  20ppm  128.600000   35.917127
3  30ppm  287.200000  363.964260
4  40ppm  583.444444  157.582547
```

Table S4. Pairwise Tukey HSD p-values for sinuosity comparisons between BPA treatments at each minute

Treatment1	Treatment2	p_value. Min 1	p_value. Min 2	p_value. Min 3	p_value. Min 4	p_value. Min 5
**0ppm**	**10ppm**	1	0.9998	1	0.9993	0.9552
**0ppm**	**20ppm**	1	0.9988	1	1	0.9997
**0ppm**	**30ppm**	0.5596	0.9754	0.9485	0.9862	0.4262
**0ppm**	**40ppm**	1	0.5511	0.6368	***0.0263***	0.1061
**10ppm**	**20ppm**	0.9999	1	1	0.9999	0.9854
**10ppm**	**30ppm**	0.5106	0.9438	0.9678	0.9987	0.7982
**10ppm**	**40ppm**	1	0.6381	0.6877	***0.0374***	0.2848
**20ppm**	**30ppm**	0.5778	0.912	0.964	0.9929	0.5232
**20ppm**	**40ppm**	1	0.6982	0.6763	***0.0298***	0.1401
**30ppm**	**40ppm**	0.5348	0.2767	0.9533	***0.0566***	0.8466

Figure S1. Some trajectories of rotifer individuals under varying BPA exposure conditions.

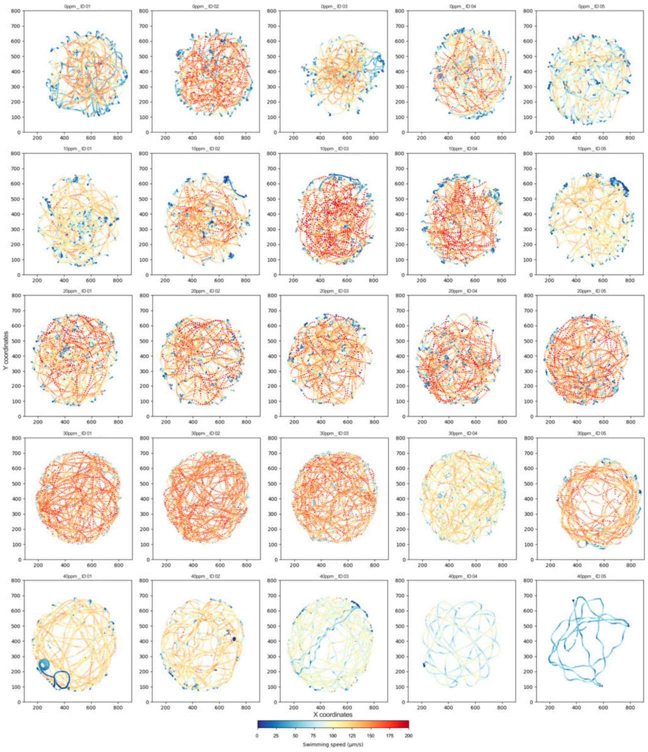

Supplement: Supplementary file 1 [file toxics-13-00723-s001.zip › toxics-3706840-supplementary.pdf]
